# Antibody and Local Cytokine Response to Respiratory Syncytial Virus Infection in Community-Dwelling Older Adults

**DOI:** 10.1128/mSphere.00577-20

**Published:** 2020-09-02

**Authors:** Xiao Yu, Anke J. Lakerveld, Sandra Imholz, Marion Hendriks, Sofie C. A. ten Brink, H. Lie Mulder, Karen de Haan, Rutger M. Schepp, Willem Luytjes, Menno D. de Jong, Josine van Beek, Puck B. van Kasteren

**Affiliations:** a Laboratory of Clinical Virology, Department of Medical Microbiology, Amsterdam University Medical Center, University of Amsterdam, Amsterdam, the Netherlands; b Center for Infectious Disease Control, National Institute for Public Health and the Environment, Bilthoven, the Netherlands; National Institute of Allergy and Infectious Diseases

**Keywords:** elderly, interferon, cytokine, mucosa, IgG, IgA

## Abstract

Respiratory syncytial virus (RSV) is increasingly recognized for causing severe morbidity and mortality in older adults, but there are few studies on the RSV-induced immune response in this population. Information on the immunological processes at play during RSV infection in specific risk groups is essential for the rational and targeted design of novel vaccines and therapeutics. Here, we assessed the antibody and local cytokine response to RSV infection in community-dwelling older adults (≥60 years of age). During three winters, serum and nasopharyngeal swab samples were collected from study participants during acute respiratory infection and recovery. RSV IgG enzyme-linked immunosorbent assays (ELISA) and virus neutralization assays were performed on serum samples from RSV-infected individuals (*n* = 41) and controls (*n* = 563 and *n* = 197, respectively). Nasal RSV IgA and cytokine concentrations were determined using multiplex immunoassays in a subset of participants. An *in vitro* model of differentiated primary bronchial epithelial cells was used to assess RSV-induced cytokine responses over time. A statistically significant increase in serum neutralization titers and IgG concentrations was observed in RSV-infected participants compared to controls. During acute RSV infection, a statistically significant local upregulation of beta interferon (IFN-β), IFN-λ1, IFN-γ, interleukin 1β (IL-1β), tumor necrosis factor alpha (TNF-α), IL-6, IL-10, CXCL8, and CXCL10 was found. IFN-β, IFN-λ1, CXCL8, and CXCL10 were also upregulated in the epithelial model upon RSV infection. In conclusion, this study provides novel insights into the basic immune response to RSV infection in an important and understudied risk population, providing leads for future studies that are essential for the prevention and treatment of severe RSV disease in older adults.

**IMPORTANCE** Respiratory syncytial virus (RSV) can cause severe morbidity and mortality in certain risk groups, especially infants and older adults. Currently no (prophylactic) treatment is available, except for a partially effective yet highly expensive monoclonal antibody. RSV therefore remains a major public health concern. To allow targeted development of novel vaccines and therapeutics, it is of great importance to understand the immunological mechanisms that underlie (protection from) severe disease in specific risk populations. Since most RSV-related studies focus on infants, there are only very limited data available concerning the response to RSV in the elderly population. Therefore, in this study, RSV-induced antibody responses and local cytokine secretion were assessed in community-dwelling older adults. These data provide novel insights that will benefit ongoing efforts to design safe and effective prevention and treatment strategies for RSV in an understudied risk group.

## Abstract (precis)

Respiratory syncytial virus (RSV) can cause severe morbidity and mortality in certain risk groups, especially infants and older adults. Currently no (prophylactic) treatment is available, except for a partially effective yet highly expensive monoclonal antibody. RSV therefore remains a major public health concern. To allow targeted development of novel vaccines and therapeutics, it is of great importance to understand the immunological mechanisms that underlie (protection from) severe disease in specific risk populations. Since most RSV-related studies focus on infants, there are only very limited data available concerning the response to RSV in the elderly population. Therefore, in this study, RSV-induced antibody responses and local cytokine secretion were assessed in community-dwelling older adults. These data provide novel insights that will benefit ongoing efforts to design safe and effective prevention and treatment strategies for RSV in an understudied risk group.

## INTRODUCTION

Although primarily known for causing severe disease in infants, respiratory syncytial virus (RSV) is increasingly recognized to cause severe morbidity and mortality in older adults (1–3). Globally, RSV acute respiratory infections in older adults were estimated to have resulted in 336,000 hospitalizations in 2015, causing an estimated 14,000 in-hospital deaths and likely many more outside the hospital setting (3). Other than a partially effective and very expensive monoclonal antibody (palivizumab), which is used only in high-risk infants (4), there are currently no vaccines or specific antivirals available for the prevention and treatment of RSV disease. The development of safe and effective strategies critically depends on a thorough understanding of the immunological mechanisms underlying (protection from) severe disease. However, these mechanisms and associated correlates of protection may not be the same in different risk groups. For example, unlike infants, older adults have already experienced multiple RSV infections throughout life and often suffer from waning immunity. Although there is a vast amount of literature available on the human immune response to RSV infection (for a review, see reference 5), most studies focus on the infant population, and only a few report data on the immune response in older adults (6–10).

While most RSV vaccination strategies that are currently being developed aim at inducing virus-specific antibodies, the extent to which these contribute to protection and their exact protective mechanisms remain uncertain (for a review, see reference 11). RSV particles contain two major surface glycoproteins: the attachment protein (G) and the fusion protein (F). In particular, antibodies targeting the prefusion form of the F protein (pre-F) appear to mediate neutralization (12), although G-specific antibodies are also likely to contribute (13). However, the neutralizing capacity of RSV-specific serum immunoglobulin G (IgG) appears not to correlate well with protection *in vivo* (8, 14). Higher titers might indicate a better chance of being protected, but a protective threshold probably does not exist (8, 14). The concentration of RSV-specific IgA in the nasal mucosa appears to correlate slightly better with protection than serum IgG level, but this response is short-lived, and again, an established protective threshold is lacking (8, 14, 15). Notably, most studies investigating the antibody response to RSV have focused on infants or nonelderly adults.

RSV primarily infects the epithelia of the upper and lower respiratory tract. Whereas the majority of individuals experience only mild symptoms upon infection, infants and elderly persons can develop severe, life-threatening disease, such as bronchiolitis and pneumonia. The mechanisms underlying severe RSV disease are incompletely understood, but a dysregulated immune response—for example, due to an immature or waning immune response—appears to be an important component (for a review, see reference 16). Notably, the production of cytokines in the respiratory mucosa is likely of crucial importance in modulating the subsequent immune response (for a review, see reference 17). Again, whereas many studies provide data on mucosal cytokine expression in infants, data specific to the older adult population are scarce (9).

In this study, antibody and local cytokine responses were assessed in RSV-infected older adults (≥60 years of age) during acute infection and recovery. RSV-specific neutralization titers and IgG concentrations were determined in serum, as well as antigen-specific IgA and cytokine concentrations in nasal samples. In addition, a time course RSV infection experiment in primary differentiated bronchial epithelial cells was performed to identify cytokines that are likely epithelium derived. Together, these data provide novel insights into the immune response to RSV in elderly individuals, which may contribute to the targeted development of preventive and therapeutic strategies for RSV in this understudied risk group.

## RESULTS

### RSV-specific serum neutralization titers and IgG concentrations increase upon infection in older adults.

**FIG 1:**
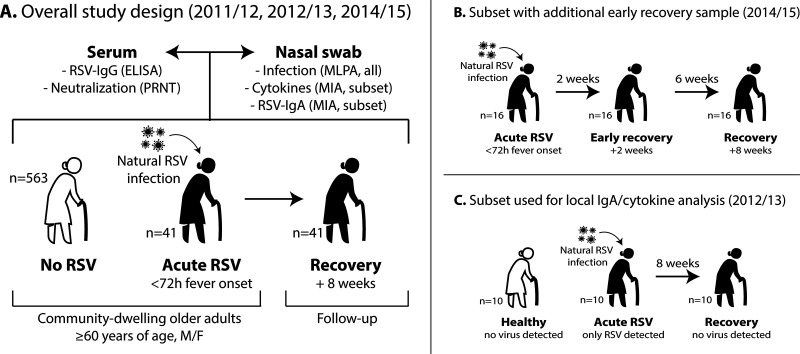
Schematic overview of the experimental design of the study. (A) The complete data set includes RSV-infected individuals (*n* = 41), from whom samples were taken during acute infection (<72 h after onset of fever) and recovery (8 weeks later). In addition, controls without RSV infection (*n* = 563) were included, some of whom were noninfected and some of whom had respiratory infections other than RSV. Serum and nasopharyngeal swabs were collected and used for various assays. (B) During the 2014-2015 season, symptomatic participants underwent an additional early-recovery sampling at 2 weeks after the acute-phase sampling. This resulted in a subset of RSV-infected individuals (*n* = 16) from whom samples were taken at three time points: acute phase, early recovery (+2 weeks), and recovery (+8 weeks). (C) To analyze the local IgA and cytokine responses to RSV infection, we selected 10 symptomatic participants with MLPA-confirmed RSV infection and no other detectable viral infections during the acute phase. All of the selected participants were negative for any respiratory viral infection during the recovery phase. As healthy controls, we selected 10 age- and sex-matched participants without symptoms who were negative for any respiratory viral infection during sampling. ELISA, enzyme-linked immunosorbent assay; IgA/IgG, immunoglobulin A/G; M/F, male/female; MIA, multiplex immunoassay; MLPA, multiplex ligation-dependent probe amplification; PRNT, plaque reduction neutralization test; RSV, respiratory syncytial virus.

**FIG 2:**
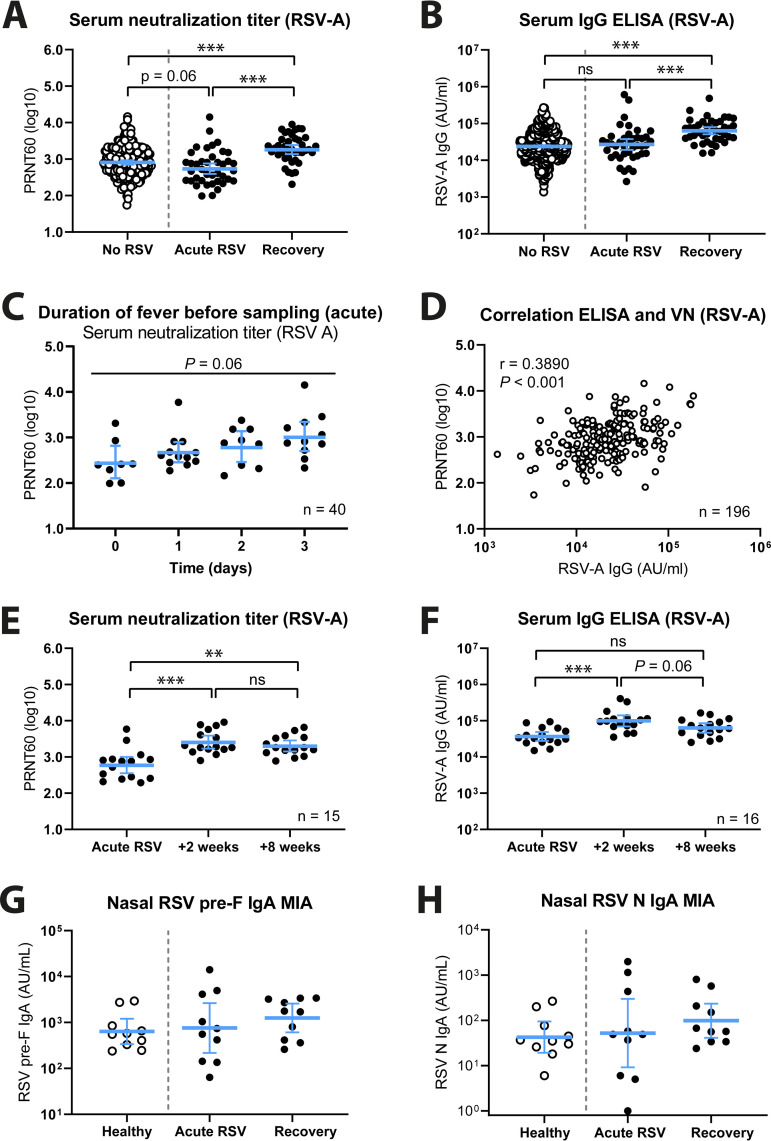
Serologic analyses of RSV-infected participants and controls. (A) Virus neutralization titers were determined by PRNT for controls without RSV (*n* = 197; white circles) and RSV-infected persons during acute infection and recovery (*n* = 40; black circles). (B) RSV-A-specific serum IgG concentrations were determined by ELISA for controls without RSV (*n* = 563; white circles) and RSV-infected persons during acute infection and recovery (*n* = 41; black circles). PRNT and log-transformed ELISA data were analyzed using an ordinary one-way ANOVA with Tukey’s multiple-comparison test. (C) Participant samples were taken <72 h after fever onset, ranging from 0 to 3 days. The plot shows acute-phase serum neutralization titers, according to the interval between fever onset and sampling. PRNT data were analyzed using an ordinary one-way ANOVA. (D) Correlation between PRNT and ELISA data for the controls without RSV (*n* = 196). Data were assessed by Pearson correlation. (E) Virus neutralization titers were determined by PRNT for RSV-infected persons (*n* = 15) during acute infection, early recovery (+2 weeks), and recovery (+8 weeks). (F) RSV-A-specific serum IgG concentrations were determined by ELISA for RSV-infected persons (*n* = 16) during acute infection, early recovery (+2 weeks), and recovery (+8 weeks). Data in panels E and F were analyzed using the Friedman test with Dunn’s multiple-comparison test. (G and H) RSV prefusion F-specific (G) and nucleoprotein (N)-specific (H) nasal IgA concentrations were determined by multiplex immunoassay in a subset of participants. All data points represent individual participants, and lines indicate geometric means and 95% confidence intervals. *, *P < *0.05; **, *P < *0.01; ***, *P < *0.001; ns, not significant. AU/mL, arbitrary units per milliliter; ELISA, enzyme-linked immune sorbent assay; IgA/IgG, immunoglobulin A/G; PRNT, plaque reduction neutralization test.

Serum neutralization capacity and IgG concentration are two of the most commonly assessed parameters when the immune response to RSV is studied. For this reason, we compared these two immunological characteristics between cases with confirmed RSV infection, during both acute infection and recovery 8 weeks later, and controls without confirmed RSV infection at any sampling during the study. For a schematic overview of the different groups that were analyzed, see Fig. 1A. Of note, in this study the no-RSV controls consisted of both individuals without respiratory infections and individuals with respiratory infections other than RSV. We found that mean serum neutralization titers were higher during the recovery phase in RSV-infected patients than during acute infection or in controls without RSV infection (Fig. 2A) and that these differences were statistically significant (*P < *0.001). Furthermore, mean neutralization titers were marginally lower in RSV-infected patients during acute infection than in controls without RSV infection (*P = *0.06). We found comparable results for RSV-specific serum IgG concentrations (Fig. 2B; Fig. S1A), except that in this case the mean IgG concentration during acute RSV infection was approximately the same as, if not slightly higher than, in controls without RSV infection. Notably, the RSV-specific neutralization titers measured during acute-phase sampling had likely already increased compared to pre-exposure levels, as an increasing trend in mean titers correlating with the interval between onset of fever and sampling can be observed (*P = *0.06) (Fig. 2C). Interestingly, we observed only a weak correlation between serum neutralization titers and RSV-specific IgG concentrations in controls without RSV infection (*r* = 0.3890, *P < *0.001) (Fig. 2D), which is in line with previous literature (8).

10.1128/mSphere.00577-20.1 FIG S1 Additional serologic analyses of RSV-infected participants and controls. (A) RSV-B-specific serum IgG concentrations were determined by ELISA for controls without RSV (*n* = 562; white circles) and RSV-infected persons during acute infection and recovery (*n* = 41; black circles). Log-transformed ELISA data were analyzed using an ordinary one-way ANOVA with Tukey’s multiple-comparison test. (B) RSV-B-specific serum IgG concentrations were determined by ELISA for RSV-infected persons (*n* = 16) during acute infection, early recovery (+2 weeks), and recovery (+8 weeks). Data were analyzed using the Friedman test with Dunn’s multiple-comparison test. (C to E) RSV-specific nasal IgA concentrations were determined by multiplex immunoassay for postfusion F, G_A_, and G_B_ in a subset of participants. All data points represent individual participants, and lines indicate geometric means and 95% confidence intervals. **, P < 0.01; ***, P < 0.001; ns, not significant. AU/mL, arbitrary units per milliliter; ELISA, enzyme-linked immunosorbent assay; IgA/IgG, immunoglobulin A/G. Download FIG S1, TIF file, 1.5 MB. Copyright © 2020 Yu et al. 2020 Yu et al. This content is distributed under the terms of the Creative Commons Attribution 4.0 International license.

For one of the cohorts of this study (2014-2015), an additional sampling time, at 2 weeks after initial acute-infection sampling, was included (Fig. 1B). Data obtained from the 16 RSV-infected patients in this cohort show that mean serum neutralization titers and RSV-specific IgG concentrations are significantly (*P < *0.01) increased at this early recovery time point compared to those during acute infection (Fig. 2E and F; Fig. S1B). Furthermore, although not statistically significant, it appears that both mean neutralization titer and IgG concentrations had started to decline at 8 weeks following acute infection compared to the levels observed after 2 weeks.

Bearing in mind that nasal RSV-specific IgA concentrations have been shown to provide a better indication of protection than serum antibodies (8, 14), we next determined antigen-specific nasal IgA concentrations using a multiplex immunoassay in a subset of 10 RSV-infected patients, during both acute infection and recovery, and 10 healthy noninfected controls (Fig. 1C). As antigens, we included both the prefusion and postfusion conformations of the F protein (pre-F and post-F, respectively), the nucleoprotein (N), and G proteins from both a group A and B strain (G_A_ and G_B_). Despite the low number of samples and large variability, we observed an apparent trend of increase in mean nasal IgA concentrations for all antigens in RSV-infected cases during recovery compared to healthy controls (Fig. 2G and H; Fig. S1C to E). The large variability in IgA concentration observed in the acute-infection group might suggest that this group consisted of a combination of individuals, some of whom had and some of whom had not (yet) responded to the infection.

### Acute-phase serum neutralization titers show a weak negative correlation with duration of coughing, which does not reach statistical significance.

**FIG 3:**
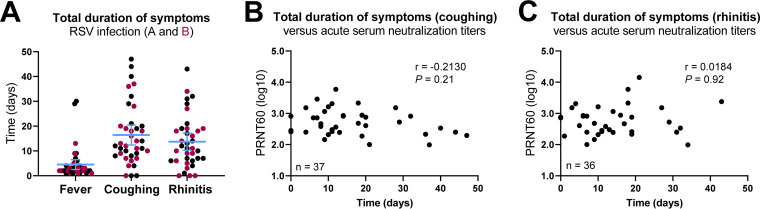
Analysis of RSV disease symptoms in relation to acute-phase serum neutralization titers. (A) Self-reported start and end dates of disease symptoms were recorded. All data points represent individual participants, and lines indicate means and 95% confidence intervals. (B and C) Plots showing the correlation between acute-phase serum neutralization titers (PRNT60) and duration of coughing (B; *n* = 37) and rhinitis (C; *n* = 36) for RSV-infected participants. For some participants, only the start date of coughing and rhinitis was recorded, and they were excluded from the analysis (*n* = 3 and *n* = 4, respectively). Data were assessed by Spearman correlation. PRNT, plaque reduction neutralization test.

During the study period, self-reported start and end dates of disease symptoms were recorded. As a criterion for sampling, all confirmed RSV-infected participants displayed a fever which lasted for 4.5 days on average (95% confidence interval [CI], 2.5 to 6.5) (Fig. 3A). Upon RSV infection, the vast majority of participants also experienced coughing and rhinitis lasting on average 16.4 (95% CI, 12.4 to 20.4) and 13.7 (95% CI, 10.3 to 17.2) days, respectively (Fig. 3A). We did not observe an apparent difference in symptom duration between RSV A and B infections (Fig. 3A). We hypothesized that acute-phase serum neutralization titers might correlate with symptom duration, but we were unable to confirm or reject this possibility with the available data (Fig. 3B and C).

### RSV infection induces upregulation of a variety of cytokines in the nasal mucosa of older adults.

Mucosal cytokine production plays an important role in modulation of the (protective or pathogenic) immune response to RSV (17). However, few data are available on the local cytokine response in the older adult population specifically (9). For this reason, we assessed the concentrations of a selection of 13 cytokines, known to be generally involved in the antiviral response, in nasal samples from a subset of participants (Fig. 1C). To restrict our findings specifically to the RSV-mediated response, we selected 10 RSV-infected individuals in whom no additional respiratory viruses were detected. In addition, these individuals tested negative for all respiratory viruses, including RSV, during recovery sampling. As healthy controls, we included 10 samples from age- and sex-matched participants without symptoms of infection who, in addition, tested negative for all respiratory viruses included in the diagnostic panel.

**FIG 4:**
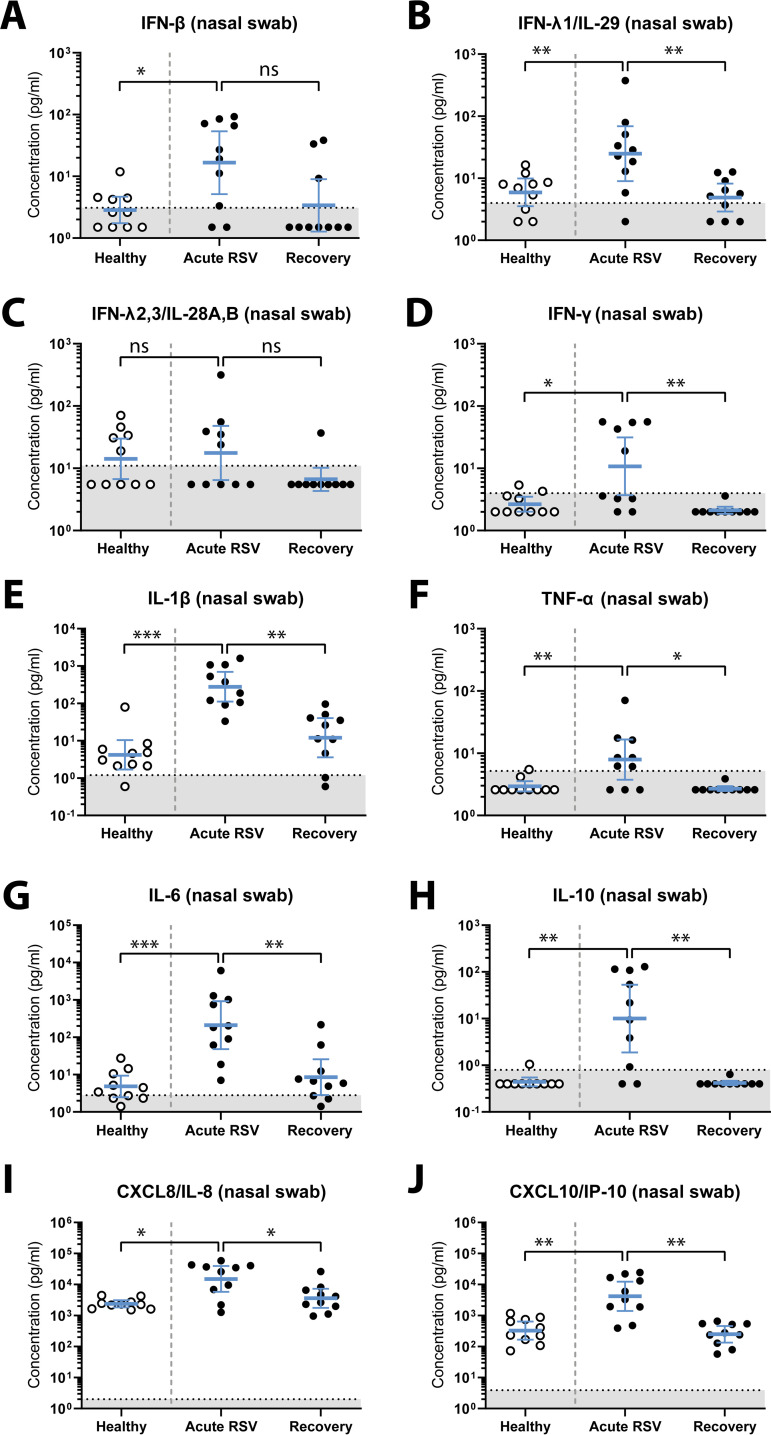
Upregulation of nasal cytokines in RSV-infected older adults. A multiplex immunoassay was used to determine the nasal concentration of IFN-β (A), IFN-λ1 (B), IFN-λ2/3 (C), IFN-γ (D), IL-1β (E), TNF-α (F), IL-6 (G), IL-10 (H), CXCL8 (I), and CXCL10 (J). Nasopharyngeal swab samples were collected from study participants within 72 h of presenting with fever (acute RSV; *n* = 10) and controls without respiratory viral infection (healthy; *n* = 10). Samples were taken from RSV-infected individuals again during recovery, 8 weeks later (recovery; *n* = 10). Data points represent individual participants, and lines indicate geometric mean concentrations and 95% confidence intervals. Measurements below the detection limit were set to 0.5 times the lower limit of detection. Unpaired samples from healthy and acutely RSV-infected individuals were compared using a nonparametric Mann-Whitney test. Paired samples from individuals during acute RSV infection and recovery were compared using a nonparametric Wilcoxon matched-pairs signed-rank test. *, *P < *0.05; **, *P < *0.01; ***, *P < *0.001; ns, not significant. Dotted lines indicate the lower limit of detection. IFN, interferon; IL, interleukin; RSV, respiratory syncytial virus; TNF, tumor necrosis factor.

We found a statistically significant increase in nasal concentrations of beta interferon (IFN-β), IFN-λ1, IFN-γ, interleukin 1β (IL-1β), tumor necrosis factor alpha (TNF-α), IL-6, IL-10, CXCL8, and CXCL10 in RSV-infected individuals during acute infection compared to healthy controls (Fig. 4 and Table 1). In addition, we observed a statistically significant decrease in the concentrations of most of these cytokines during follow-up (recovery) in previously RSV-infected persons compared to acute infection, except for IFN-β. We did not find statistically significant differences between groups in the nasal concentration of IFN-λ2/3 (Fig. 4C and Table 1), and we were unable to detect IFN-α2, granulocyte-macrophage colony-stimulating factor (GM-CSF), and IL-12p70 in any of the groups (Fig. S2A). Notably, the lack of detection of these cytokines does not necessarily imply their absence *in vivo*, since this might also be explained by cytokine-specific issues with sample handling, storage, or analysis.

**TABLE 1:** Nasal cytokine concentrations in RSV-infected individuals (acute RSV) and noninfected controls (healthy and recovery)^*a*^^,^^*b*^

Cytokine	Group	GMC^*c*^ (pg/ml)	CI (95%)	*P* value^*d*^
IFN-β	Healthy	2.8	1.7–4.7	0.03
	Acute RSV	17	5.1–54	NA
	Recovery	3.4	1.3–9.0	0.13
IFN-λ1	Healthy	5.9	3.5–9.9	0.009
	Acute RSV	25	9.0–69	NA
	Recovery	4.9	2.9–8.2	0.01
IFN-λ2/3	Healthy	14	6.7–30	0.80
	Acute RSV	18	6.5–48	NA
	Recovery	6.7	4.3–10	0.13
IFN-γ	Healthy	2.7	2.0–3.5	0.04
	Acute RSV	11	3.7–31	NA
	Recovery	2.1	1.9–2.4	0.008
IL-1β	Healthy	4.2	1.7–10	<0.001
	Acute RSV	278	111–696	NA
	Recovery	12	3.6–40	0.002
TNF-α	Healthy	2.9	2.4–3.6	0.007
	Acute RSV	7.9	3.8–17	NA
	Recovery	2.7	2.5–3.0	0.02
IL-6	Healthy	4.8	2.5–9.4	<0.001
	Acute RSV	210	48–919	NA
	Recovery	8.6	2.8–26	0.002
IL-10	Healthy	0.44	0.35–0.55	0.001
	Acute RSV	10	1.9–53	NA
	Recovery	0.42	0.38–0.47	0.008
CXCL8	Healthy	2,382	1,828–3,103	0.009
	Acute RSV	15,120	5757–39,715	NA
	Recovery	3,590	1,769–7,285	0.04
CXCL10	Healthy	326	167–639	0.001
	Acute RSV	4,169	1,405–12,366	NA
	Recovery	249	135–459	0.004

a Each group consisted of 10 individuals. The acute and recovery groups are paired measurements from the same individuals, and the healthy controls are age- and sex-matched with the RSV-infected cases. Abbreviations: CI, confidence interval; GMC, geometric mean concentration, NA, not applicable.

b In addition to the cytokines included in this table, we measured IFN-α2, GM-CSF, and IL-12p70. However, the concentrations of these cytokines were below the assay limit of detection.

c For calculation purposes, values that were below the detection limit were set at 0.5 times the lower limit of detection.

d The difference between acute and recovery was assessed using a nonparametric Wilcoxon matched-pairs signed-rank test, and the difference between acute and healthy was assessed using an unpaired nonparametric Mann-Whitney test. A *P* value of <0.05 was considered statistically significant.

10.1128/mSphere.00577-20.2 FIG S2 Cytokines that remained below the limit of detection or were unchanged upon RSV infection. (A) A multiplex immunoassay was used to determine the concentration of IFN-α2, GM-CSF, and IL-12p70 in nasal samples. Nasopharyngeal swab samples were collected from study participants within 72 h of presenting with fever (acute RSV; *n* = 10) and controls without respiratory viral infection (healthy; *n* = 10). Samples were taken from RSV-infected individuals again during recovery, 8 weeks later (recovery; *n* = 10). Data points represent individual participants, and lines indicate geometric mean concentrations and 95% confidence intervals. Measurements below the detection limit were set to 0.5 times the lower limit of detection. Dotted line indicates the lower limit of detection. (B) A multiplex immunoassay was used to determine the concentrations of IFN-α2, IFN-γ, IL-1β, TNF-α, IL-6, IL-10, GM-CSF, and IL-12p70 in basolateral medium and apical wash samples of mock- or RSV-infected HAE cultures. Samples were collected from 12 to 336 h postinfection from mock- and RSV-infected HAE cultures derived from three individual donors. Values are geometric mean concentrations and standard deviations. Measurements below the detection limit were set to 0.5 times the lower limit of detection. Dotted lines indicate the lower limit of detection. Download FIG S2, TIF file, 2.5 MB. Copyright © 2020 Yu et al. 2020 Yu et al. This content is distributed under the terms of the Creative Commons Attribution 4.0 International license.

### RSV infects ciliated cells in primary differentiated HAE cultures.

**FIG 5:**
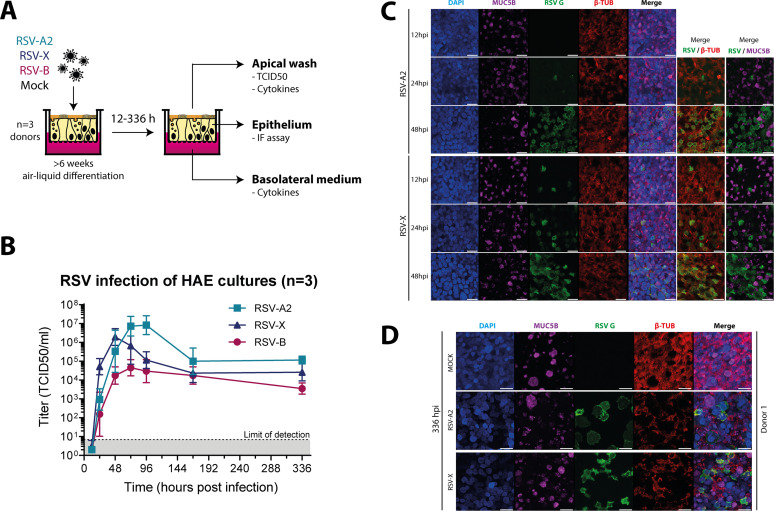
RSV infection of primary human airway epithelial (HAE) cultures at the air-liquid interface. (A) Schematic representation of the experimental setup. (B) Titration (TCID_50_) of apical wash samples collected 12 to 336 h postinfection from RSV-infected HAE cultures derived from three individual donors during two independent experiments. Lines indicate geometric mean titers with standard deviations. (C and D) HAE cultures were infected with RSV-A2 or RSV-X or mock infected, as indicated, and incubated for 12, 24, and 48 h (C) and 336 h (D). Goblet cells, RSV-infected cells, and ciliated cells were subsequently visualized by staining for mucin 5B (MUC5B; purple), RSV G protein (green), and β-tubulin (β-TUB; red), respectively. Nuclei are stained with DAPI (blue). Images are from one representative donor. Bars, 20 μm.

To further elucidate the mucosal cytokine response to RSV infection, we used *in vitro* air-liquid interface primary human airway epithelial (HAE) cultures to dissect the innate epithelium-specific cytokine response. We inoculated HAE cultures with two strains of RSV-A (RSV-A2 and RSV-X) and one RSV-B strain (WA/18537/62) and collected samples at various time points (12 to 336 h). For a schematic representation of the experimental setup, see Fig. 5A. We first confirmed successful infection of the HAE cultures by performing a virus titration assay on a time course of apical wash samples (Fig. 5B). The three strains displayed similar growth curves, reaching peak titers at 48 to 72 h postinfection (hpi). RSV-B infection resulted in the lowest peak titers, reflecting either lower replication efficiency or limited release of virus particles from the epithelial cell layer. Virus replication ultimately reached a stable phase with continued production of infectious viral particles up to at least 14 days postinfection (336 hpi). In addition to titration, we performed immunofluorescence microscopy to assess spread of the infection in HAE cultures for RSV-A2 and RSV-X. For this, epithelia were stained for RSV infection (G protein; green), ciliated cells (β-tubulin; red), and mucus-producing goblet cells (mucin 5B; purple). We observed a gradual increase in the number of infected cells from 12 to 48 hpi for both virus strains (Fig. 5C; Fig. S3A). As previously described, RSV infection was limited to ciliated cells (18, 19). Consistent with the observed persistence of production of infectious virus, infected cells could still be found at 336 hpi (Fig. 5D; Fig. S3B). Whereas RSV infection did not appear to greatly affect epithelial structural integrity up to 48 hpi, we did observe syncytium formation and a clear decrease in ciliation at 336 hpi.

10.1128/mSphere.00577-20.3 FIG S3 Immunofluorescence staining of RSV-infected HAE cultures. (A) In parallel with the RSV infections whose results are depicted in Fig. 4, HAE cultures were mock infected and incubated for 12, 24, and 48 h. (B) HAE cultures were infected with RSV-A2 or RSV-X or mock infected, as indicated, and incubated for 336 h. Goblet cells, RSV-infected cells, and ciliated cells were subsequently visualized by staining for mucin 5B (MUC5B; purple), RSV G protein (green), and β-tubulin (β-TUB; red), respectively. Nuclei are stained with DAPI (blue). Images in panel A are from the donor represented in Fig. 4; images in panel B are from two additional donors. Bars, 20 μm. Download FIG S3, JPG file, 2.2 MB. Copyright © 2020 Yu et al. 2020 Yu et al. This content is distributed under the terms of the Creative Commons Attribution 4.0 International license.

### RSV induces expression of a limited number of cytokines in differentiated airway epithelial cultures.

**FIG 6:**
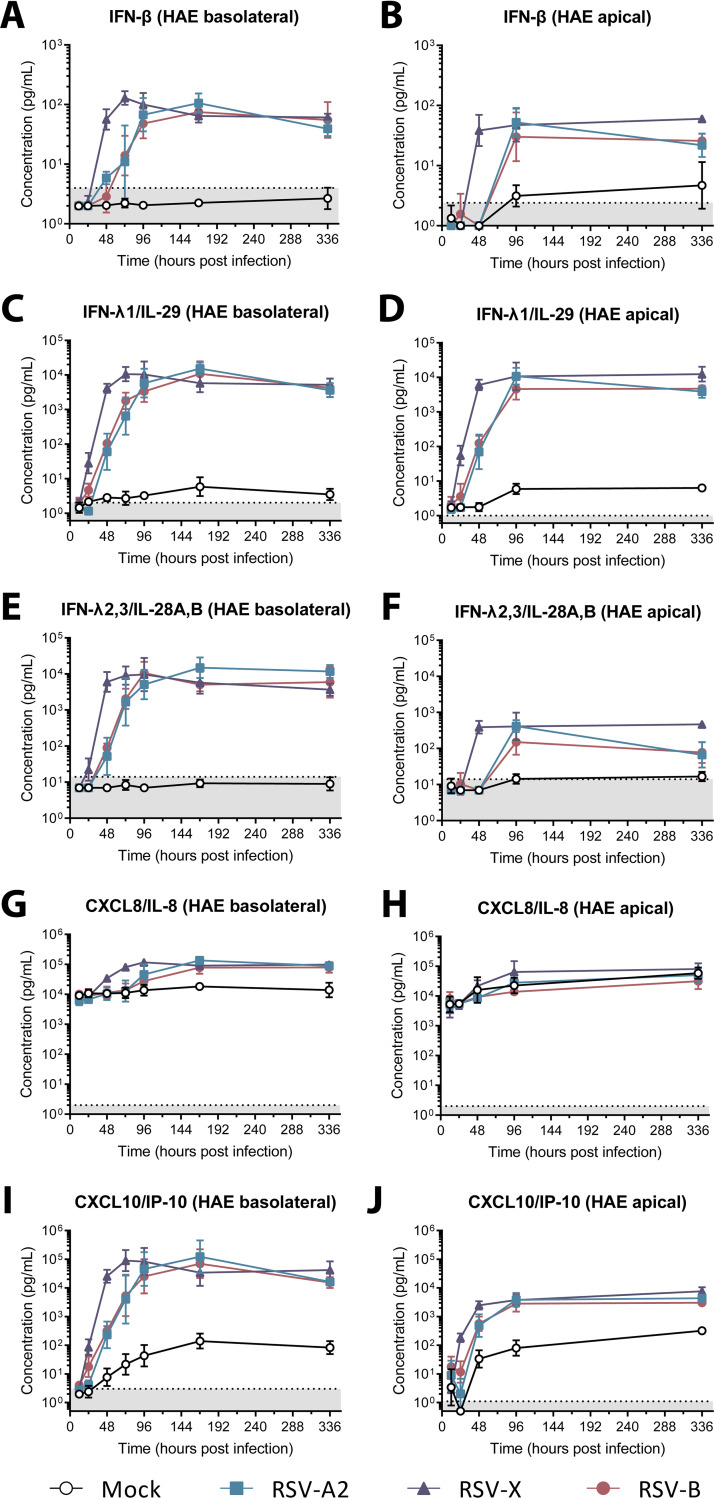
Upregulation of cytokines in basolateral medium and apical wash samples of RSV-infected HAE cultures. A multiplex immunoassay was used to determine the concentration of IFN-β (A and B), IFN-λ1 (C and D), IFN-λ2/3 (E and F), CXCL8 (G and H), and CXCL10 (I and J). Basolateral medium and apical wash samples were collected from 12 to 336 h postinfection from mock- and RSV-infected HAE cultures derived from three individual donors in two independent experiments. Values are geometric mean concentrations and standard deviations. Measurements below the detection limit were set to 0.5 times the lower limit of detection. Dotted lines indicate the lower limit of detection. HAE, human airway epithelial; IFN, interferon; RSV, respiratory syncytial virus.

Finally, we used both basolateral medium and apical wash samples of RSV-infected HAE cultures at different times postinfection to assess the concentrations of the cytokine panel used for nasal samples. We found a marked upregulation of IFN-β, IFN-λ1, IFN-λ2/3, CXCL8, and CXCL10 in basolateral medium samples upon RSV infection compared to mock infection, starting from 24 to 48 hpi (Fig. 6). These concentrations reached a plateau around 96 hpi and remained stable up to 336 hpi. With the exception of CXCL8, we observed a similar RSV-mediated response in the apical wash samples, although the difference from mock infection was generally less pronounced than that observed for the basolateral medium samples. Of note, unlike the other cytokines that were measured, basal levels of CXCL8 were very high, and an increase compared to mock-infected cultures was observed only in the basolateral medium samples, not in apical wash samples (Fig. 6G and H). Although overall, the three virus strains showed a similar pattern of cytokine induction, the RSV-X-induced response was consistently slightly ahead in time of that induced by RSV-A2 and RSV-B. This earlier response appears to correlate with a slightly more efficient replication and/or spread of RSV-X than RSV-A2 and RSV-B in the first days of infection (Fig. 5B and C). Following RSV infection, the concentrations of IFN-α2, IFN-γ, IL-1β, TNF-α, IL-6, IL-10, GM-CSF, and IL-12p70 in basolateral medium and apical wash samples were below the limit of detection or did not notably differ from those in mock-infected cultures (Fig. S2B).

## DISCUSSION

Since most studies investigating the immune response to RSV infection in humans focus on infants, there is only limited information available on the immune response, either protective or pathogenic, to RSV in the older adult population. In the present study, we show that RSV infection in community-dwelling older adults (≥60 years of age) induces an increase in serum RSV neutralization titers and RSV-specific IgG concentration. Furthermore, we found that RSV infection in this population results in an upregulation in the nasal mucosa of IFN-β, IFN-λ1, IFN-γ, IL-1β, TNF-α, IL-6, IL-10, CXCL8, and CXCL10. Finally, in an *in vitro* model of differentiated primary human bronchial epithelial cells, we observed RSV-mediated upregulation of IFN-β, IFN-λ1, IFN-λ2/3, CXCL8, and CXCL10.

Previous studies have shown that pre-exposure serum RSV neutralization titers and RSV-specific IgG concentrations do not correlate very well with protection from infection (8, 14). While apparently susceptible individuals tend to display slightly lower titers and concentrations than those who do not become infected, there is probably no threshold level above which one is surely protected from infection. Due to the lack of baseline samples for RSV-infected participants in our study, we were unable to corroborate that susceptible individuals had lower pre-exposure titers than those who were supposedly protected, i.e., controls without RSV infection. However, considering that we observed an increase in serum neutralization titers depending on the interval between onset of fever and sampling and considering existing data from older adults showing that serum RSV neutralization titers start to increase within 1 to 3 days from the onset of disease (9), it is likely that in our study, pre-exposure serum RSV neutralization titers in RSV-infected participants were in fact slightly lower than those in controls. It thus seems that (neutralizing) antibodies do contribute, at least in part, to protection from RSV infection in older adults, but future work should also take into account the role of cellular immunity as an additional means of protection.

An important factor complicating the assessment of studies like the one presented here is the fact that due to its observational nature, the control group likely consists of individuals with variable exposure to RSV. For this reason, it is likely that some of the control subjects had not become infected with RSV not because of their immune status but merely because they had not been exposed to the virus. The use of experimental and controlled human infection models would circumvent this problem, but out of ethical considerations, such studies cannot be performed with older adults, who are generally at increased risk for severe complications. Another option would be to focus studies on older adults with frequent exposure to young children, as RSV exposure in this group will likely be more common. RSV-specific antibody neutralization titers and concentrations appeared to have started to decrease at 8 weeks after acute infection. For future studies, it would be interesting to perform a longer follow-up to further characterize the dynamics and longevity of the RSV-specific antibody response in older adults.

As shown in this study and others, all adults possess RSV-specific serum antibodies which have various capacities for virus neutralization (8, 14). When these virus-specific antibodies are not able to completely prevent infection, as often appears to be the case, they might still be able to mitigate the development of severe disease, for example, via the recruitment and activation of (innate) immune cells through Fc-mediated interactions (for reviews, see references 11 and 20). We have recently shown that, in infants, the capacity of RSV-specific antibodies to activate natural killer cells might contribute to protection from severe disease (21). In contrast, these antibodies potentially also contribute to the development of severe disease, in a process referred to as antibody-dependent enhancement (ADE) of infection and/or inflammation. Future studies in older adults, including patients with various degrees of RSV disease severity and baseline samples, are needed to elucidate the mechanisms by which RSV-specific antibodies prevent, mitigate, and/or contribute to severe RSV pathology in this particular population. We found a weak correlation between serum IgG concentration and neutralization titers, which has also been described by others (8). Such observations highlight the importance of including functional assays (e.g., neutralization and Fc-mediated functionality) in studies of the antibody response to RSV. Whereas concentration is of course an important factor, antibody functionality also strongly depends on isotype and subclass composition, antigen and epitope specificity, and Fc glycosylation. Characterizing the antibody response in its broadest sense, by means of a comprehensive systems serology approach, will likely provide important insights into the role of RSV-specific antibodies in protection against RSV infection and disease (22).

In addition to the antibody response, we assessed mucosal cytokine concentrations in nasal samples of older adults and found that several were upregulated in an apparently RSV-dependent manner. Whereas an association between nasal IL-6 concentration and RSV disease severity in the adult population has previously been described (9), to our knowledge the upregulation of IFN-β, IFN-λ1, IFN-γ, IL-1β, TNF-α, IL-10, CXCL8, and CXCL10 upon RSV infection in the older adult population specifically has not been demonstrated before. Notably, most of these cytokines have previously been shown to have a (beneficial and/or detrimental) role in RSV-mediated disease in children (for a review, see reference 5). In this young population, a protective role has been described for IFN-γ (23–25), while a variable association with disease severity was found for IL-6 (26–28) and IL-10 (28, 29), and a deleterious role has been suggested for IL-1β (26, 27), TNF-α (26), CXCL8 (26, 27), and IFN-λ (30). Further studies are needed to assess the role of these cytokines in RSV pathogenesis in older adults, for example by comparing patients with various degrees of disease severity.

In this study, we found that IFN-β, IFN-λ1, CXCL8, and CXCL10 levels are elevated both *in vivo* and *in vitro*, highlighting the potential of the HAE culture model in recapitulating the *in vivo* inflammatory response. As the epithelial model has a less complex cellular composition than the nasal mucosa, we found upregulation of fewer cytokines in the *in vitro* model than *in vivo*. For example, we did not detect IL-1β *in vitro*, whereas it was one of the cytokines with the most pronounced upregulation *in vivo*. Indeed, RSV-mediated expression of this cytokine in mice has been shown to require the presence of neutrophils and macrophages (31).

Our *in vitro* findings largely recapitulate what has previously been found in comparable differentiated epithelial models which were mainly derived from children: upregulation of CXCL8, CXCL10, and IFN-λ1 (32–35), while IL-1β and TNF-α are not expressed (34). Notably, previous studies investigating the response to RSV infection in undifferentiated epithelial cells did report upregulation of IL-1β, TNF-α, and IFN-γ (36–38), highlighting some of the differences between these models. Whereas several studies previously described an RSV-induced upregulation of IL-6 in differentiated epithelial cells (32–34), we did not observe marked changes in IL-6 secretion in our *in vitro* model. Furthermore, we observed a clear induction of IFN-β and IFN-λ2/3 secretion, which was not found in earlier studies (33, 34). The use of different RSV strains (39) and methods for culturing and/or analysis might account for these discrepancies. Strikingly, in contrast to our *in vitro* findings, we did not observe an RSV-dependent upregulation of IFN-λ2/3 *in vivo*. A possible explanation for this difference is that our *in vitro* model is derived from bronchial cells, while *in vivo*, we examined the nasal mucosa. Indeed, previous studies have reported differences in cytokine expression levels between bronchial and nasal epithelial cells (33). It would be of particular interest to conduct single-cell sequencing of nasal brushes from RSV-infected individuals to decipher the exact cellular origin of the various cytokines *in vivo* and to elucidate how various *in vitro* epithelial models compare to the *in vivo* situation. Additionally, *in vitro* coculture models of differentiated airway epithelial and immune cells will likely provide novel insights on their interaction and resulting cytokine expression profiles.

In conclusion, our study provides novel insights into the immune response to RSV infection in the older adult population. Future work in older adults should aim at elucidating the contribution of antibody (Fc-mediated) functionality to protection, while also taking into account the role of cellular immunity. Studies comparing RSV patients with various degrees of disease severity are needed to establish the roles of cytokines, antibody functionality, and cellular immunity in (protection from) progression to severe disease. Taken together, such studies will provide the basis for the rational design of targeted strategies for the prevention and treatment of RSV infection in a high-risk and understudied population.

## MATERIALS AND METHODS

### Study design.

Clinical samples were obtained as part of a prospective observational cohort study performed in the Netherlands during the winters of 2011-2012, 2012-2013, and 2014-2015 to assess the occurrence of influenza-like illness (ILI) in community-dwelling older adults (40). Participants aged ≥60 years were recruited through their general practitioner or the civil registry. No exclusion criteria were used. Written informed consent was obtained from all participants. All trial-related activities were conducted according to good clinical practice, which includes the provisions of the Declaration of Helsinki. The study was approved by the acknowledged ethical committee METC Noord-Holland (http://www.trialregister.nl; NL3234 and NL4666).

Participants were asked to report the occurrence of ILI according to the Dutch Pel criteria, defined by fever (≥37.8°C) with at least one other symptom among the following: headache, myalgia, sore throat, coughing, rhinitis, or chest pain (41). Within 72 h of fever onset, i.e., during the acute phase, a research nurse collected samples during a home visit. A second sampling was performed 8 weeks (±1 week) later during the recovery phase. In the 2014-2015 cohort, an additional sampling was performed 2 weeks after the acute phase. Self-reported start and end dates of disease symptoms were recorded. As healthy controls, samples from subjects without symptoms of infection participating in the same cohort study were taken throughout the season. Participant characteristics for all groups and subsets can be found in Table S1. There were no statistically significant differences in age or sex between groups.

10.1128/mSphere.00577-20.4 TABLE S1 Participant characteristics. a, The “no RSV” controls consisted of both individuals without infection and individuals with respiratory infections other than RSV. b, The ages of 2 individuals were not recorded, and they were excluded from the characteristics analysis; a single participant was 59 years old. c, The sex of 16 individuals was not recorded, and they were excluded from the characteristics analysis. d, Nonparametric Mann-Whitney test. e, Fisher’s exact test. f, No respiratory viruses were detected in samples from these individuals. g, No respiratory viruses other than RSV were detected in samples from these individuals during the acute phase, and no respiratory viruses, including RSV, were detected in these individuals during recovery. Download Table S1, PDF file, 0.1 MB. Copyright © 2020 Yu et al. 2020 Yu et al. This content is distributed under the terms of the Creative Commons Attribution 4.0 International license.

### Sampling and diagnostics.

Serum samples were collected in serum collection tubes with clot activator and gel separator, aliquoted within 8 h, and stored at −80°C. Nasopharyngeal samples were obtained with a sterile swab with a flocked nylon tip and stored in 1 ml modified liquid Amies transport medium (Eswab; Copan, Brescia, Italy). Swab samples were transported at room temperature to the laboratory, where the samples were vortexed, aliquoted, and stored at −80°C within 8 h after sampling. Diagnostics were performed on all nasopharyngeal swabs by a multiplex ligation-dependent probe amplification (MLPA) assay for a broad panel of respiratory viral pathogens, including influenza A and B viruses, RSV A and B, human metapneumovirus, rhino/enterovirus, adenovirus, parainfluenza viruses 1 to 4, bocavirus, and coronavirus NL63/OC43/229E/HKU1 (RespiFinder Smart 22; PathoFinder, Maastricht, the Netherlands).

### Cell lines and viruses.

Vero cells (ATCC CCL-81) were cultured in Dulbecco’s modified Eagle’s medium (DMEM; Gibco). HEp2 cells (ATCC CCL-23) were cultured in minimum essential medium (MEM; Gibco). All media were supplemented with 10% heat-inactivated fetal bovine serum (HyClone, Fisher Scientific) and 1% penicillin, streptomycin, and glutamine (Gibco).

Human RSV-A2 (ATCC VR-1540) was propagated on HEp2 cells. RSV-98-25147-X (referred to here as X; GenBank FJ948820), RSV-X-GFP7 (42), and RSV-B/WA/18537/62 (referred to here as B) were propagated on Vero cells. Virus stocks used for infection experiments were purified by polyethylene glycol precipitation (A2) or by ultracentrifugation between layers of 10% and 50% sucrose (X and B). Virus titers were determined by 50% tissue culture infective dose (TCID_50_) assay on Vero cells according to the Spearman and Karber method (43) and converted to PFU per milliliter by multiplying by 0.69.

### Serologic assays.

RSV-specific serum IgG concentrations were determined by enzyme-linked immunosorbent assay (ELISA). Immulon plates were coated with RSV-A2 (inactivated with 2% Triton X-100) or purified RSV-B. After blocking with 2% bovine serum albumin (BSA) in phosphate-buffered saline (PBS), plates were incubated with sera diluted 1:1,500 in PBS containing 0.1% Tween 80. After washing, plates were incubated with horseradish peroxidase (HRP)-conjugated polyclonal rabbit anti-human IgG (Dako), and HRP activity was assessed using TMB (3,3′,5,5-tetramethylbenzidine) Single Solution (Life Technologies) on an ELISA plate reader. For this analysis, we included acute and recovery samples from all RSV-infected participants (*n* = 41), a selection of samples (either acute cases or healthy controls) from participants without confirmed RSV at any sampling during the study (*n* = 563), and the additional 2-week samples from RSV-infected participants in the 2014-2015 cohort (*n* = 16).

Virus neutralization titers were determined by the plaque reduction neutralization test (PRNT) using RSV-X-GFP7, as described before (42). Samples included in this analysis were essentially the same as for the ELISA, except that PRNT data were not recorded or measured for one RSV-infected individual, and a lower number of participants without RSV were included as controls (*n* = 197).

RSV-specific IgA concentrations were determined in nasal samples by a multiplex immunoassay, essentially as described before (44). Fluorescent beads (Bio-Rad Laboratories) were coated with RSV pre-F, post-F, N, G_A_, or G_B_ protein, and bound IgA was detected using goat F(ab')2 anti-human IgA conjugated to phycoerythrin (PE) (Southern Biotech). For this, we selected 10 symptomatic participants with MLPA-confirmed RSV infection and no other detectable viral infections during the acute phase. All of the selected participants were negative for viral infections during the recovery phase. As healthy controls, we selected 10 age- and sex-matched participants without symptoms who were negative for viral infection during sampling.

### HAE cultures at the air-liquid interface.

Primary human airway epithelial cells were obtained from patients undergoing lung lobectomy at Amsterdam University Medical Center (A-UMC). Signed informed consent was obtained from all patients before sampling. The study was performed under EU regulations and was approved by the Institutional Review Board of the A-UMC (2015_122#A2301550). Healthy epithelial cells from the excised tissue were isolated and cultured following the protocol of Fulcher and Randell (45). For this study, cells from three different donors (1 male and 2 females; 56, 57, and 71 years old) were used.

Primary human airway epithelial cells were initially expanded in PneumaCult-ExPlus medium (number 05040; Stemcell) in T75 flasks coated with type I collagen (VitroCol, 5007-20ML; Advanced Biomatrix). When 80 to 90% confluence was reached, cells were trypsinized into single-cell suspensions and transferred in PneumaCult-ExPlus medium to 0.4-μm-pore-size Transwell inserts (number 3470; Corning) coated with type IV collagen (C7521-10MG; Sigma). When confluence was reached, apical media were removed and basolateral media were replaced with PneumaCult-ALI medium (number 05001; Stemcell) to initiate differentiation at the air-liquid interface.

### Infection.

Following 6 weeks of differentiation, 1 × 10^5^ PFU RSV-A2, -X, or -B in a total volume of 200 μl Hanks’ balanced salt solution (HBSS) was added to the apical side of the HAE inserts, while HBSS alone was used for mock infection. After 2 h of incubation at 37°C, the inoculum was removed and the apical side was washed twice with HBSS. At various time points, the apical side of the epithelium was sampled by washing with 200 μl HBSS for 20 min at 37°C. Basolateral media were sampled by collecting 200 μl medium and replenishing. Apical and basolateral samples were snap-frozen and stored at −80°C until further analysis. For immunofluorescence staining, cells were fixed in 4% formaldehyde for 20 min at room temperature. Inserts were washed and stored in Dulbecco’s PBS at 4°C.

### Immunofluorescence staining.

Fixed HAE cultures were permeabilized in 0.1% Triton X-100 for 1 h, followed by blocking for 30 min in 1% BSA in PBS. Rabbit anti-MUC5B (sc-20119; Santa Cruz) and mouse anti-RSV-G (clone 021/19G) (46) were incubated for 2 h, followed by 1 h of incubation with donkey anti-rabbit IgG–Cy5 (711-175-152; Jackson) and donkey anti-mouse IgG–A488 (A21202; Life Technologies) and a final incubation for 1 h with anti-β-tubulin–Cy3 (C4585; Sigma). The inserts were embedded in VectaShield with 4′,6-diamidino-2-phenylindole (DAPI; H-1500; Vector Laboratories). Pictures were obtained using a Leica SP8 confocal microscope. Maxi-projection was performed on the z-stack planes.

### Cytokine analysis.

Cytokine concentrations in the nasal samples of selected individuals (the subset used for local IgA measurements [Fig. 1C]), HAE apical wash samples, and HAE basolateral medium samples were determined using the human antivirus response panel LEGENDplex (number 740390; BioLegend), according to the manufacturer’s instructions. This assay contained the following targets: IL-1β, IL-6, IL-8/CXCL8, IL-10, IL-12-p70, IFN-α2, IFN-β, IFN-γ, IFN-λ1/IL-29, IFN-λ2,3/IL-28A,B, CXCL10/IP-10, GM-CSF, and TNF-α.

### Statistics.

All data obtained from clinical samples are presented as individual data points with geometric means and 95% confidence intervals. PRNT and log-transformed ELISA data were analyzed using an ordinary one-way analysis of variance (ANOVA) with Tukey’s multiple-comparison test. Correlation between PRNT and ELISA data was assessed by Pearson correlation. Correlation between PRNT and symptom duration was assessed by Spearman correlation. Data obtained for the subset of participants with a 2-week sample were analyzed using the Friedman test with Dunn’s multiple-comparison test. For the cytokine data, unpaired samples from healthy and acutely RSV-infected individuals were compared using a nonparametric Mann-Whitney test, and paired samples from individuals during acute RSV infection and recovery were compared using a nonparametric Wilcoxon matched-pairs signed-rank test. A *P* value of <0.05 was considered statistically significant. Statistical analysis was performed with GraphPad Prism 8.2.1 software. All data obtained from HAE cultures are graphed as geometric mean concentrations with standard deviations.
