# Supplementary figures and images for: Antibody and Local Cytokine Response to Respiratory Syncytial Virus Infection in Community-Dwelling Older Adults
Source: mSphere. 2020 Sep 2;5(5):e00577-20. doi: 10.1128/mSphere.00577-20 (PMC7471002; doi:10.1128/mSphere.00577-20)

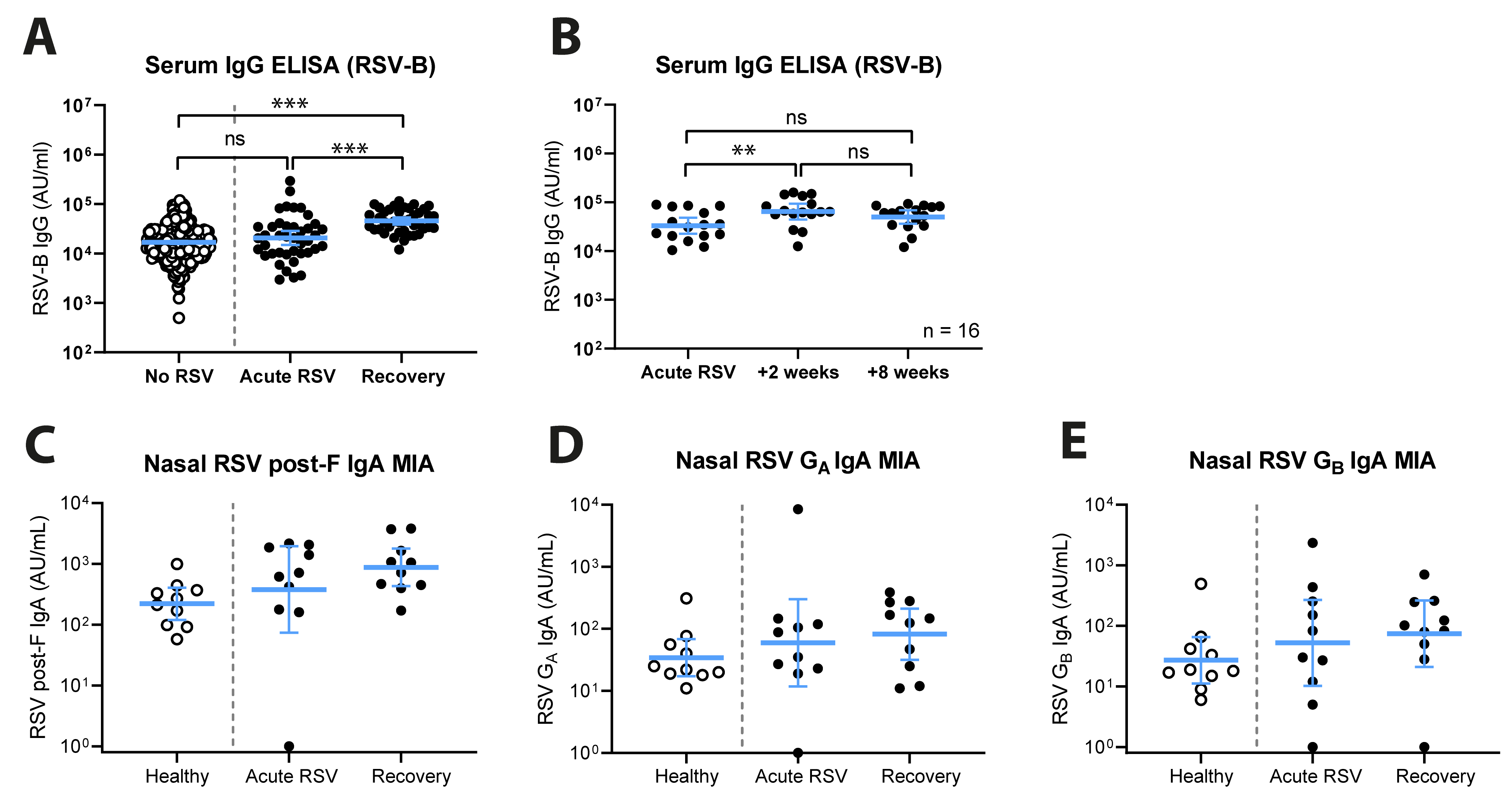

Supplement: FIG S1 [file mSphere.00577-20-sf001.tif]

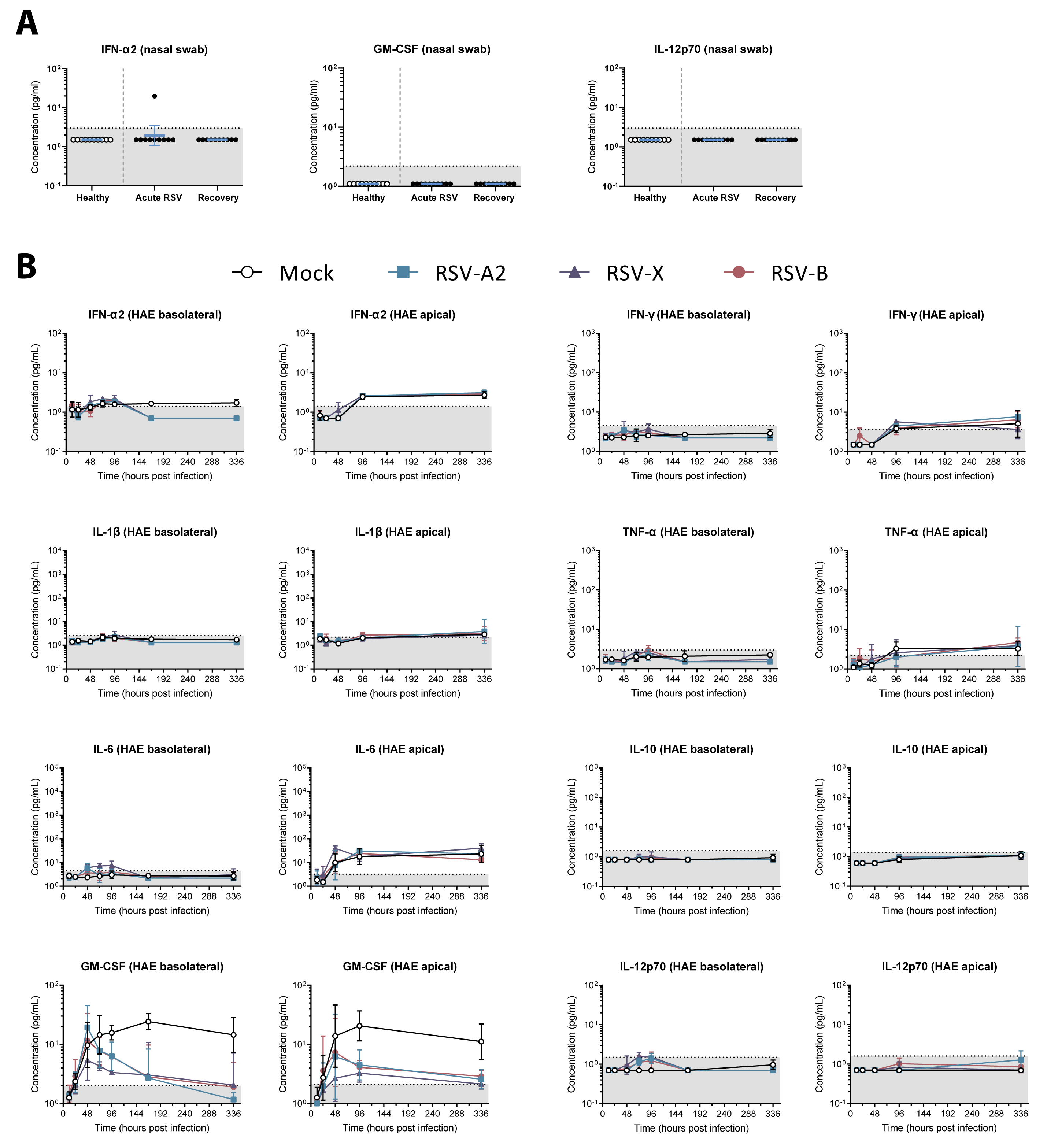

Supplement: FIG S2 [file mSphere.00577-20-sf002.tif]

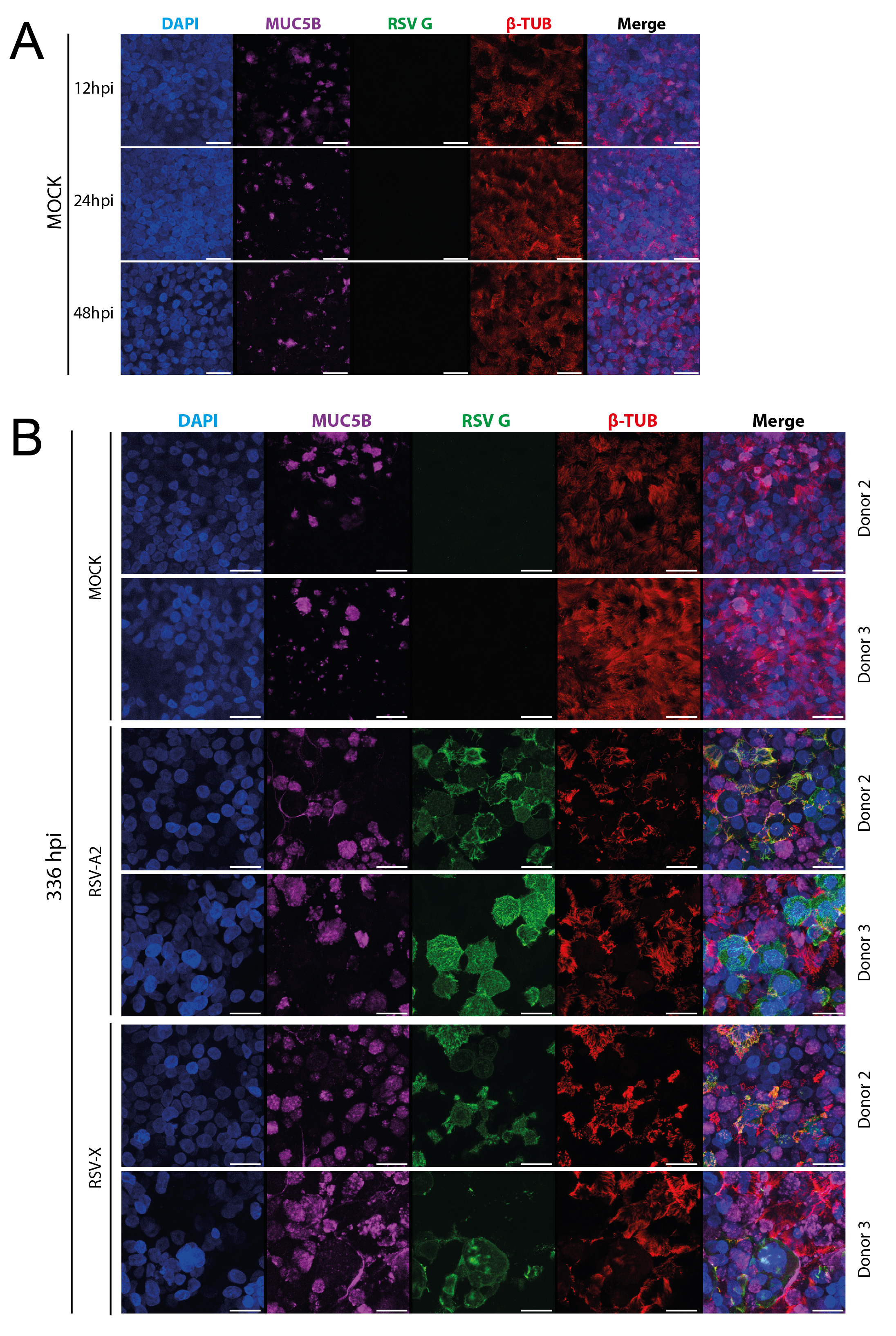

Supplement: FIG S3 [file mSphere.00577-20-sf003.jpg]
