# Supplementary material for: Antibody and Local Cytokine Response to Respiratory Syncytial Virus Infection in Community-Dwelling Older Adults
Source: mSphere. 2020 Sep 2;5(5):e00577-20. doi: 10.1128/mSphere.00577-20 (PMC7471002; doi:10.1128/mSphere.00577-20)
Supplement: TABLE S1 [file mSphere.00577-20-st001.pdf]

Overall participants (MLPA, ELISA) - cohorts 2011/12, 2012/13, 2014/15 (Fig. 2B, 3, and S1A)			
	**No RSV[a]**	**RSV-infected**	***P*-value**
Number (n)	563	41	NA
Age in years, mean (range)	69.1 (59-89)[b]	70.2 (60-88)	0.51[d]
Female sex, number (%)	293 (54)[c]	23 (56)	0.87[e]
RSV A, number (%)	0 (0)	21 (51)	NA
RSV B, number (%)	0 (0)	20 (49)	NA
**Subset tested for virus neutralization - cohorts 2011/12, 2012/13, 2014/15 (Fig. 2A,C,D)**			
	**No RSV[a]**	**RSV-infected**	***P*-value**
Number (n)	197	40	NA
Age in years, mean (range)	69.4 (59-89)[b]	70.2 (60-88)	0.51[d]
Female sex, number (%)	111 (56)	22 (55)	>0.99[e]
RSV A, number (%)	0 (0)	21 (53)	NA
RSV B, number (%)	0 (0)	19 (48)	NA
**Subset with additional early recovery sample - cohort 2014/15 (Fig. 2E-F, and S1B)**			
		**RSV-infected**	
Number (n)		16	
Age in years, mean (range)		71.8 (62-88)	
Female sex, number (%)		10 (63)	
RSV A, number (%)		7 (44)	
RSV B, number (%)		9 (56)	
**Subset used for local IgA/cytokine analysis - cohort 2012/13 (Fig. 2G-H, 4, S1C-E, and S2A)**			
	**Healthy[f]**	**RSV-infected[g]**	***P*-value**
Number (n)	10	10	NA
Age in years, mean (range)	68.6 (62-82)	68.3 (61-81)	0.93[d]
Female sex, number (%)	5 (50)	5 (50)	>0.99[e]
RSV A, number (%)	0 (0)	7 (70)	NA
RSV B, number (%)	0 (0)	3 (30)	NA
